# Supplementary figures and images for: Measuring single-cell susceptibility to antibiotics within monoclonal bacterial populations
Source: PLoS One. 2024 Aug 1;19(8):e0303630. doi: 10.1371/journal.pone.0303630 (PMC11293721; doi:10.1371/journal.pone.0303630)

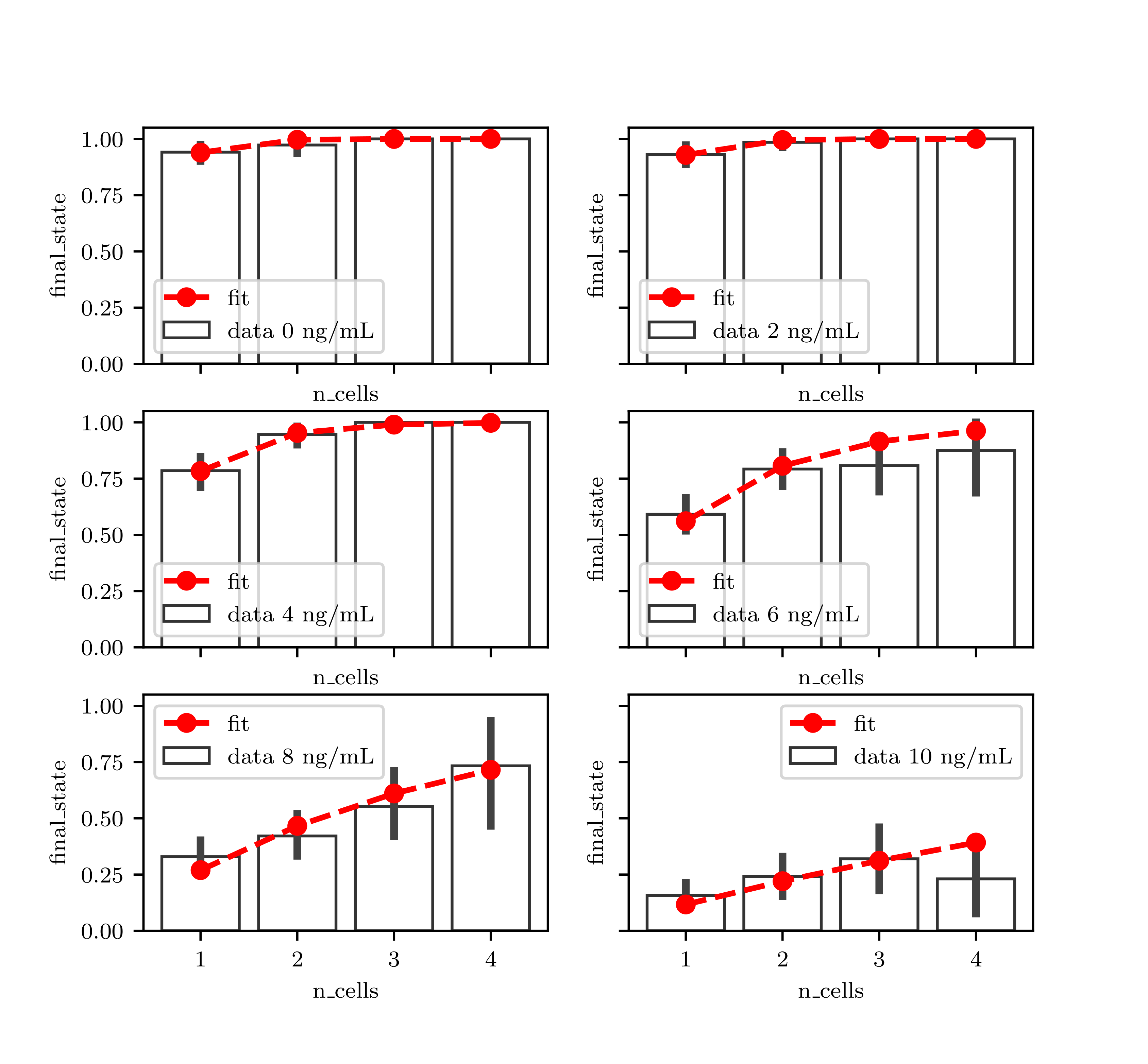

Supplement: S1 Fig — (TIFF) [file pone.0303630.s001.tiff]

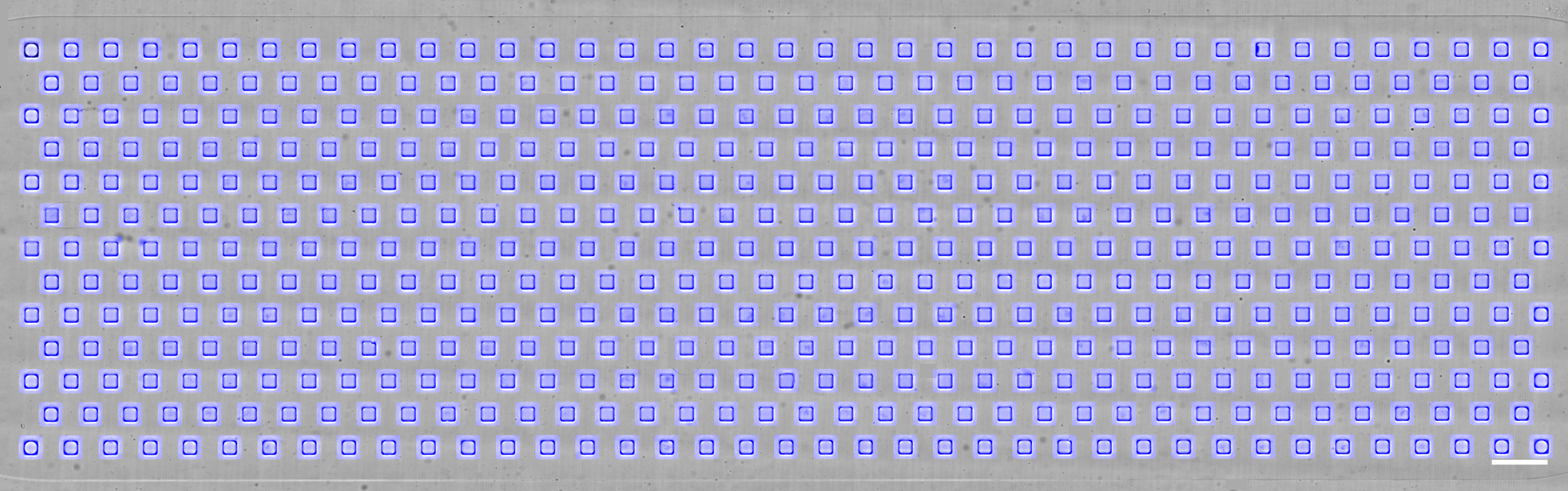

Supplement: S2 Fig — Scalebar 500 um. (TIFF) [file pone.0303630.s002.tiff]
